# Risk-taking and pathological gambling behavior in Huntington’s disease

**DOI:** 10.3389/fnbeh.2014.00103

**Published:** 2014-04-02

**Authors:** Carla Kalkhoven, Cor Sennef, Ard Peeters, Ruud van den Bos

**Affiliations:** ^1^Chardon Pharma, HerpenNetherlands; ^2^Department of Organismal Animal Physiology, Faculty of Science, Radboud University NijmegenNijmegen, Netherlands

**Keywords:** Huntington’s disease, risk-taking, gambling, prefrontal cortex, basal ganglia, disinhibtion

## Abstract

Huntington’s disease (HD) is a genetic, neurodegenerative disorder, which specifically affects striatal neurons of the indirect pathway, resulting in a progressive decline in muscle coordination and loss of emotional and cognitive control. Interestingly, predisposition to pathological gambling and other addictions involves disturbances in the same cortico-striatal circuits that are affected in HD, and display similar disinhibition-related symptoms, including changed sensitivity to punishments and rewards, impulsivity, and inability to consider long-term advantages over short-term rewards. Both HD patients and pathological gamblers also show similar performance deficits on risky decision-making tasks, such as the Iowa Gambling Task (IGT). These similarities suggest that HD patients are a likely risk group for gambling problems. However, such problems have only incidentally been observed in HD patients. In this review, we aim to characterize the risk of pathological gambling in HD, as well as the underlying neurobiological mechanisms. Especially with the current rise of easily accessible Internet gambling opportunities, it is important to understand these risks and provide appropriate patient support accordingly. Based on neuropathological and behavioral findings, we propose that HD patients may not have an increased tendency to seek risks and start gambling, but that they do have an increased chance of developing an addiction once they engage in gambling activities. Therefore, current and future developments of Internet gambling possibilities and related addictions should be regarded with care, especially for vulnerable groups like HD patients.

## Introduction

Huntington’s disease (HD) is a genetic neurodegenerative disorder, inherited in an autosomal dominant fashion. The disease is characterized by progressive motor, cognitive and behavioral symptoms, which usually become apparent between 30 and 50 years of age, and lead to premature death in 10–20 years after disease onset. HD is caused by a mutation in the Huntingtin gene (HTT), which leads to protein aggregation, deregulation of several cellular processes, and eventually cell death. Neuronal degeneration initially occurs selectively in the striatum (caudate nucleus and putamen), where it affects cortico-striatal pathways that serve to control motor and cognitive functions (Reiner et al., 2011; Vonsattel et al., 2011). At the motor level, this degenerative process is expressed as disorganized movements (chorea), while at the cognitive/behavioral level patients display an “executive dysfunction syndrome”, encompassing amongst others impulsivity, poor risk assessment and an inability to halt a poor course of action (Hamilton et al., 2003; Duff et al., 2010b). Similar behavioral and cognitive symptoms are seen in addictive behavior related to substances or activities (Newman, 1987; Rosenblatt, 2007; Iacono et al., 2008). Therefore, it may be expected that HD patients are at risk of developing addictions. Decision-making paradigms in laboratory settings have indeed suggested deficits in risky decision-making in advanced HD patients (e.g., Stout et al., 2001), and pathological gambling has incidentally been observed in this patient group (De Marchi et al., 1998). However, these findings are rare, and surprisingly few studies have directly examined symptoms and consequences of, for instance, behavioral disinhibition in HD.

In this review we will argue that HD patients may be a risk group for developing problematic gambling. Firstly, problematic gambling is characterized by subjects’ inability to stop gambling despite financial, personal or professional problems. Based on neurobiological disturbances and behavioral symptoms the capacity to stop gambling behavior seems diminished or absent in HD patients. Secondly, due to the more liberal attitudes towards gambling and increasing possibilities of legal and illegal Internet gambling (see e.g., Griffiths, 2003), we may expect the occurrence of gambling problems to increase in the coming years. Increased accessibility may specifically pose a risk to vulnerable groups, such as HD patients, that have not been previously exposed to such risks.

In general, changing external conditions and treatment methods can have unexpected and undesirable effects on patient behavior, especially in complex neurological diseases. Such effects are easily missed when behavioral symptoms are not regularly reevaluated. This may be best illustrated by the case of Parkinson’s disease, where the introduction of drug treatment with dopamine agonists led to impulse control disorders such as compulsive gambling, shopping, eating, and hypersexuality, caused by overstimulation of the mesolimbic dopaminergic system (Dodd et al., 2005; Witjas et al., 2012; Weintraub et al., 2013). However, these side effects were not recognized until years after the introduction of dopamine agonist therapies in combination with societal changes related to (the availability of) shopping, food consumption, sexuality, Internet, and gambling. This example illustrates that reassessment of risk factors is important to be able to provide effective treatment and guidance to patients in face of a changing environment.

Here, we will explore the disease profile of HD in relation to addiction, gambling problems, and decision-making deficits. In Section *HD: Neuropathology, Symptoms, and Progression*, progression of HD symptoms will be discussed in relation to disturbances in cortico-striatal circuits involved in task learning, sensitivity to punishment, and cognitive/impulse control. In Section *Risk Taking and Pathological Gambling Behavior in HD*, the neurobiological profile of HD patients will be discussed in the context of gambling and well-established risk-taking and decision-making tests, such as the Iowa Gambling Task (IGT) and the Cambridge Gambling Task (CGT). In Section *Discussion*, we will discuss how a characterization of gambling risks may lead to recommendations for HD patients and their caretakers on how to deal with this issue and which situations are best avoided. We also aim to identify yet unanswered questions, which may act as a starting point for future research into the occurrence and risks of gambling problems in HD patients.

## HD: neuropathology, symptoms, and progression

### Neurobiological disease mechanisms

HD is caused by an unstable CAG (trinucleotide; cytosine-adenine-guanine) repeat in the coding region of the HTT gene, which leads to the production of mutant huntingtin protein (Htt) with an expanded polyglutamine (polyQ) stretch (MacDonald et al., 1993). The number of trinucleotide repeats is inversely correlated to the age of onset of disease (Snell et al., 1993; Stine et al., 1993). The majority of HD patients has 40–55 repeats which causes typical adult-onset disorder, while expansions of more than 70 repeats lead to juvenile onset disorder. Individuals with fewer than 35 CAG repeats in the HTT gene will not develop HD. Although the exact mechanisms of HD pathogenesis remain unknown and cannot be discussed here in detail, they involve the formation of protein aggregates by polyQ expanded Htt, as well as the interaction of mutant Htt with numerous proteins that are involved in energy metabolism, protein and vesicle transport, and regulation of gene transcription (Li and Li, 2004; Jones and Hughes, 2011). The resulting deregulation of these cellular processes eventually leads to neuronal degeneration through mechanisms involving excitotoxicity and apoptosis.

**Figure 1:**
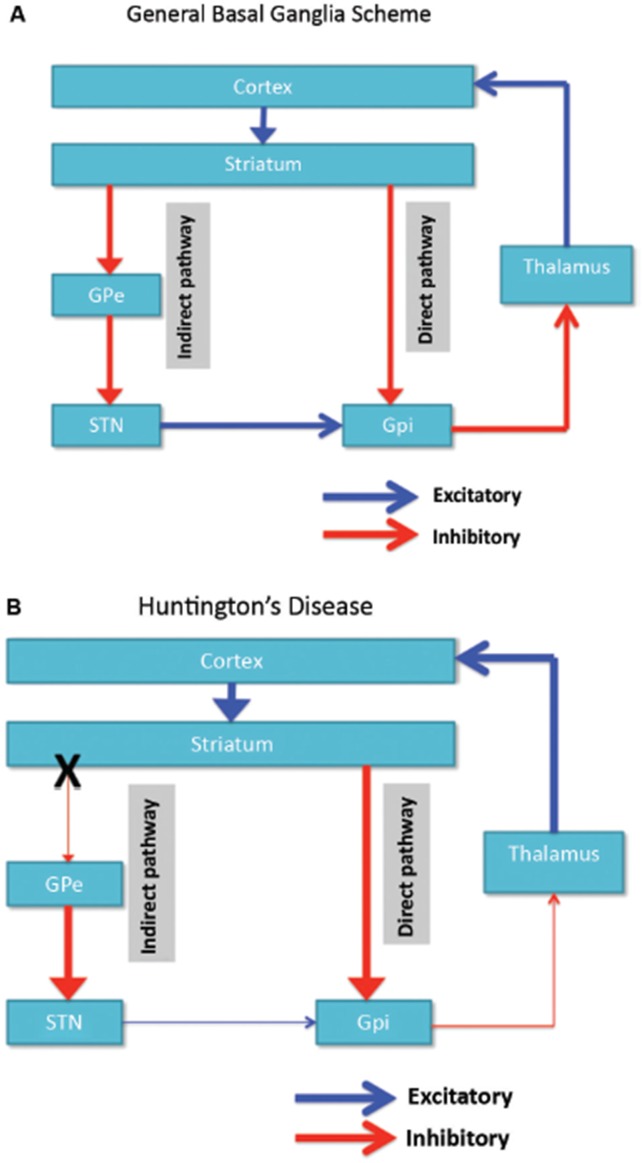
**(A)** Simplified scheme of the organization of cortico-basal ganglia networks (cortical, striatal, pallidal and thalamic areas) showing the direct and indirect pathways in normal brains. **(B)** Specific degeneration of the indirect pathway (X) in HD leads to a decrease in inhibitory control over cortical functions. GPe: external globus pallidus; GPi: internal globus pallidus; STN: subthalamic nucleus. Red: inhibitory (GABA) pathways, Blue: excitatory (glutamate) pathways.

Neuronal degeneration is initially restricted to the basal ganglia, where the medium spiny neurons in the striatum (caudate nucleus and putamen) are specifically affected (Vonsattel and DiFiglia, 1998; Kassubek et al., 2004). The striatum receives its main excitatory (glutamatergic) input from cortical areas, while it receives its dopaminergic input from the substantia nigra. The striatum has two main inhibitory (GABA-ergic) outputs: a direct and an indirect pathway (Figure 1A). Striatal neurons of the direct pathway project to the internal globus pallidus (GPi), which in turn has inhibitory projections to the thalamus. The thalamus gives rise to the main excitatory input to the cortex. Thus, in effect, activation of the direct striatal pathway inhibits GPi activity, which in turn disinhibits thalamocortical activity, thereby facilitating movement and cognitive functions. The indirect striatal pathway, on the other hand, projects to the external GP (GPe), which in turn sends inhibitory projections to the subthalamic nucleus (STN). The STN sends excitatory projections to the GPi. Accordingly, activation of the indirect striatal pathway thereby disinhibits the STN, allowing it to activate the GPi, which in turn inhibits thalamocortical activity, suppressing movement and cognitive functions. Adaptive behavior results from a (delicate) balance of activity in the direct and indirect pathway. Pathology in the indirect pathway is key to HD and disrupts the balance in striatal control resulting in a loss of inhibitory control over motor functioning and behavior (Figure 1B; Albin et al., 1989; Alexander and Crutcher, 1990).

Cortico-basal ganglia circuits, encompassing connections between cortical areas, striatal areas, pallidal areas and thalamic areas, are organized in a parallel fashion subserving different functions in the organization of behavior. As many excellent reviews exist on the anatomy and function of these circuits (e.g., Alexander et al., 1986, 1990; Alexander and Crutcher, 1990; Yin and Knowlton, 2006; Verny et al., 2007; Yin et al., 2008; Haber and Knutson, 2010; Sesack and Grace, 2010), we only highlight a few issues here conducive to our review. First, roughly speaking a dorsal to ventral topographical organization in both cortical and striatal areas exists. Thus, the dorsal prefrontal areas are associated with dorsal striatal areas while the more ventral prefrontal areas are associated with more ventral striatal areas (including the nucleus accumbens). Second, broadly three functionally different circuits may be described. The sensorimotor circuit encompasses the sensorimotor striatum (putamen) and sensorimotor cortices associated with the execution of motor behavior. The associative/cognitive control circuit involves the dorsolateral prefrontal cortex, anterior cingulate cortex, and associative striatum (caudate nucleus). This circuit is especially relevant for executive functioning, i.e., it is involved in cognitive control, planning and working memory. In addition it is involved in promoting long-term adaptive behavior by reinforcing or stopping (punishing) instrumental behavior, i.e., sequences of behavioral acts, learned in interaction with the environment (Kravitz et al., 2012; Paton and Louie, 2012). The limbic circuit includes the orbitofrontal cortex, ventromedial prefrontal cortex, amygdala, and limbic striatum (nucleus accumbens). This circuit is especially relevant for evaluating the affective value of stimuli, signaling the expected reward or punishment of an upcoming stimulus, choice or event, emotional control, and adaptive (emotional) learning (O’Doherty et al., 2001; Rushworth et al., 2007; van den Bos et al., 2013b, 2014).

Pathology in HD is observed in both the putamen and caudate nucleus (Vonsattel and DiFiglia, 1998; Kassubek et al., 2004; Vonsattel, 2008; Vonsattel et al., 2011; Hadzi et al., 2012). In addition, in both structures atrophy follows a characteristic pattern, starting in the dorsal and caudal regions and moving towards the ventral and rostral regions as the disease progresses (Vonsattel and DiFiglia, 1998; Kassubek et al., 2004; Vonsattel, 2008). However early atrophy has also been observed in the nucleus accumbens and globus pallidus in some studies (van den Bogaard et al., 2011; Sánchez-Castañeda et al., 2013). While disturbances in the sensorimotor circuit (putamen) may be related to the motor symptoms, disturbances in the associative/cognitive control circuit (caudate nucleus) may be related to executive dysfunction, and cause deficits in e.g., working memory in early HD patients (Lawrence et al., 1996; Bonelli and Cummings, 2007; Wolf et al., 2007). Disturbances in the limbic circuit, such as due to early atrophy in the nucleus accumbens, may be related to apathy and depression (Bonelli and Cummings, 2007; Unschuld et al., 2012). Progressive atrophy in the striatum may lead to a successive dysfunction of cortico-striatal circuits. For instance, the ventral caudate nucleus is also part of the orbitofrontal circuit, which is affected as the disease progresses. Dysfunction of this circuit is related to behavioral disinhibition (Bonelli and Cummings, 2007). Eventually, degeneration may spread to other brain areas, including other parts of the basal ganglia (pallidal areas and thalamus), hippocampus, amygdala and cortical areas at the late stages of the disease.

In sum, HD is characterized by a specific degeneration of striatal neurons belonging to the indirect pathway. As the disease progresses, atrophy of the striatum spreads along a caudal-rostral and dorsal-ventral gradient causing a sequential disturbance of cortico-striatal circuits. The resulting loss of inhibitory control in these circuits is directly related to the progression of motor, cognitive and behavioral symptoms in HD, as discussed below.

### Symptoms of HD

HD is characterized by a variety of progressive motor, cognitive and behavioral symptoms. The first symptoms usually arise at mid-age, with an average onset age of 40, although a small percentage of patients suffer from juvenile-onset HD, which starts before the age of 20. As the symptoms and progression of juvenile-onset HD are somewhat distinct from adult-onset disorder, we will focus on the latter patient group in this review. One of the first symptoms to become apparent in HD is chorea (involuntary movement disorder), and a clinical diagnosis is usually made after onset of movement abnormalities (Shannon, 2011). Some studies, however, report subtle cognitive and emotional changes before onset of motor symptoms, and the exact order of occurrence and progression of HD symptoms remains a subject of debate. Nevertheless, several comprehensive reviews of the clinical manifestations of HD are available (Roos, 2010; Anderson, 2011; Shannon, 2011).

#### Motor symptoms

Motor symptoms start to become apparent in the early stages of HD, and are usually the first symptoms to be noticed in laboratory settings and by first-degree relatives of HD patients (de Boo et al., 1997; Kirkwood et al., 1999, 2001). Motor disturbances appear to begin as a dysfunction in error feedback control (Smith et al., 2000), consistent with the role of the cortico-striatal motor circuit in sensorimotor learning and control (Graybiel et al., 1994). The first signs of motor abnormalities are often subtle involuntary movements (chorea) of e.g., facial muscles, fingers and toes (“twitching”), hyperreflexia, and exaggerated voluntary movements (Young et al., 1986; Shannon, 2011), which lead to a general appearance of restlessness and clumsiness in early HD patients. These abnormal movements are subtle and often go unnoticed at first, but gradually worsen and spread to all other muscles over time. Other early motor symptoms include slow or delayed saccadic eye movements (Peltsch et al., 2008) and dysarthria (Ramig, 1986; Young et al., 1986). Dysarthria, a motor speech disorder, leads to difficulty with articulation and slurring of words, which makes speech progressively more difficult to understand. Dysphagia (swallowing difficulties) is observed in most patients with an onset at mid-disease stages, and gradually worsens until patients can no longer eat unassisted and often require a feeding tube in late-stage HD (Heemskerk and Roos, 2011). Other, non-choreic motor symptoms that usually become apparent at mid-stage disease include complex gait disorder, postural instability, and dystonia (involuntary muscle contractions that cause slow repetitive movements and abnormal postures), which is often accompanied by frequent falls (Koller and Trimble, 1985; Tian et al., 1992; Louis et al., 1999; Grimbergen et al., 2008). Rigidity and bradykinesia (slowness of movement and reflexes) are sometimes observed, but are mostly restricted to cases of juvenile-onset HD (Bittenbender and Quadfasel, 1962; Hansotia et al., 1968). These motor symptoms are consistent with dysfunction of the sensorimotor (and associative/cognitive control) cortico-striatal circuits that are commonly affected in HD.

#### Behavioral and psychiatric symptoms

Behavioral disorders in HD can be complex and difficult to classify, and their occurrence and onset is highly variable between individuals. Moreover, it can sometimes be difficult to distinguish behavioral disorders from normal coping with a distressing disease (Caine and Shoulson, 1983). The number of studies that have characterized behavioral symptoms in HD is limited, and as a result there is relatively little insight in their prevalence in the disease (van Duijn et al., 2007). The most frequently and consistently reported behavioral and emotional symptoms in HD are irritability, apathy, and depression, which occur with a prevalence of approximately 50% (Caine and Shoulson, 1983; Folstein and Folstein, 1983; Craufurd et al., 2001; Kirkwood et al., 2001; van Duijn et al., 2007, 2014; Tabrizi et al., 2009). Both irritability and apathy are sometimes observed in pre-manifest HD patients (Tabrizi et al., 2009; van Duijn et al., 2014), and also depression has been reported at early clinical stages (Shiwach, 1994; Julien et al., 2007; Epping et al., 2013). These affective symptoms are among the first non-motor symptoms to be noticed by first-degree relatives (Kirkwood et al., 2001). Typical apathy-related symptoms, which gradually become worse during the course of the disease, include lack of energy, motivation and initiative, decreased perseverance and quality of work, impaired judgment, poor self-care and emotional blunting (Craufurd et al., 2001; Kirkwood et al., 2001). Depressive symptoms have been related to increased activity in the ventromedial prefrontal cortex (Unschuld et al., 2012). Irritability is associated with orbitofrontal circuit dysfunction, which leads to decreased control over emotional responses in the amygdala (Klöppel et al., 2010).

Other, less commonly observed psychiatric symptoms and disorders in HD are anxiety, obsessive-compulsive disorder, mania, schizophrenia-like psychotic symptoms, such as paranoia, hallucinations, and delusions (Caine and Shoulson, 1983; Folstein and Folstein, 1983; Craufurd et al., 2001; Kirkwood et al., 2001; van Duijn et al., 2007). These symptoms usually don’t occur until mid or late stages of the disease, although they have incidentally been reported to occur in preclinical HD patients (Duff et al., 2007). Obsessive-compulsive disorder has been associated with damage to the orbitofrontal cortex and anterior cingulate cortex, while schizophrenia, a disorder which involves deficits in organizing, planning and attention, is related to dorsolateral prefrontal cortex dysfunction (Tekin and Cummings, 2002).

It is suggested that most psychiatric symptoms in HD are in fact part of a broad, ill-defined “frontal lobe syndrome” or “executive dysfunction syndrome”, which includes symptoms such as apathy, irritability, disinhibition, impulsivity, obsessiveness, and perseveration (Lyketsos et al., 2004; Rosenblatt, 2007), all of which are commonly observed in HD patients (Hamilton et al., 2003; Duff et al., 2010b). Taken together, the literature indicates that onset and progression of behavioral symptoms in HD is heterogeneous, with affective disorders occurring most often and with early onset, while anxiety, obsessive-compulsive disorder, and psychotic symptoms are less common and usually occur later in the disease. These psychiatric symptoms are associated with dysfunction of limbic and associative/cognitive control cortico-striatal circuits that are commonly affected in HD.

#### Cognitive symptoms

Cognitive decline is another important aspect of HD pathology. Many studies have focused specifically on the occurrence of cognitive symptoms in preclinical and early clinical stages of HD, in the hope to discover early clinical biomarkers of the disease (reviewed in Papp et al., 2011; Dumas et al., 2013). Overall, results suggest that subtle cognitive changes may be observed up to 5–10 years before onset of motor symptoms with sufficiently sensitive methods. One study even found that, at preclinical and early clinical stages of HD, about 40% of patients already meet the criteria for mild cognitive impairment (a disorder associated with limited memory loss, not meeting the criteria for diagnosis of dementia; Duff et al., 2010a). However, not all studies support these findings (Blackmore et al., 1995; Giordani et al., 1995; de Boo et al., 1997; Kirkwood et al., 2001). In general, the literature agrees that information processing and psychomotor speed are especially affected at this early stage (Rothlind et al., 1993; Kirkwood et al., 1999; Verny et al., 2007; Paulsen et al., 2008). Other commonly observed early cognitive impairments include problems with attention, (working) memory, and visuospatial performance (Jason et al., 1988; Rothlind et al., 1993; Foroud et al., 1995; Lawrence et al., 1996; Hahn-Barma et al., 1998; Verny et al., 2007; Paulsen et al., 2008; Tabrizi et al., 2009; Papp et al., 2011; Stout et al., 2011). Cognitive inflexibility has been observed in early disease patients (Jason et al., 1988), at which stage extra-dimensional shifts are specifically impaired, while reversal learning is still intact (Lawrence et al., 1996). Thus, patients are still able to reevaluate stimulus value and learn new stimulus-reward contingencies within the same dimension (e.g., shape or color), but have problems shifting their attention to a different dimension (e.g., from color to shape) as required by the new task rule to obtain reward. In later stages of the disease, cognitive inflexibility and perseveration also cause impaired reversal learning in HD patients (Josiassen et al., 1983; Lange et al., 1995). This progression of symptoms is consistent with specific dysfunction of the dorsolateral prefrontal circuit early in the disease, since extra-dimensional set shifting is mediated by the dorsolateral prefrontal cortex, while reversal learning is mediated by the orbitofrontal cortex (Dias et al., 1996; McAlonan and Brown, 2003). Other early impairments include disorganized behavior, impaired planning, poor judgment, and reduced behavioral and emotional control (Watkins et al., 2000; Paradiso et al., 2008; Duff et al., 2010b). Disinhibition has been observed in early HD patients, whose performance is impaired on tasks that require inhibition of pre-potent but inappropriate responses (Holl et al., 2013). Finally, several studies have found that preclinical HD patients are impaired in the recognition of negative emotions such as anger, disgust, fear and sadness. Emotional recognition declines progressively, and can spread to problems with neutral emotions in early clinical stages of the disease (Johnson et al., 2007; Tabrizi et al., 2009; Labuschagne et al., 2013). This phenotype is related to dysfunction of the orbitofrontal cortex, which is involved in processing emotional and reward information (Henley et al., 2008; Ille et al., 2011).

Studies with animal models of HD show similar cognitive impairments to those observed in human patients. Although not all studies find robust cognitive deficits (Fielding et al., 2012), findings in rat and mouse models of HD include anxiety, increased responsiveness to negative emotional stimuli, and impairments in reversal learning and strategy shifting (Faure et al., 2011; Abada et al., 2013). One study found specific early deficits in reversal learning before onset of motor symptoms in a rat model of HD (Fink et al., 2012). Interestingly, HD animals appear to have an increased responsiveness to negative emotional stimuli, while human patients show decreased recognition of negative emotions. At present it is unclear whether this reflects differences in task administered (recognizing emotions *versus* behavioral responses to threatening stimuli), species-related differences in the outcome of pathology or a fundamental difference between the rat model and the human condition. In general, studies in both human patients and animal models of HD demonstrate that a wide range of cognitive functions can already be impaired in early HD. Early abnormalities mainly include deficits in attention, memory, cognitive flexibility, and emotional recognition. At this early stage, patients often have impaired awareness of their own (decline in) cognitive abilities (Hoth et al., 2007). Over time, cognitive symptoms progressively get worse, eventually leading to severe subcortical dementia in late stages of the disease. Although the occurrence of symptoms is generally consistent with successive impairment of associative/cognitive control and limbic cortico-striatal circuits, respectively, specific functions that are related to the limbic circuit can also already be affected at early-stage HD.

#### Conclusion

Motor, behavioral and cognitive symptoms in HD have been studied extensively in the past, and continue to be a topic of interest due to the wide variety and variability in the occurrence and onset of these symptoms across patients. In general, behavioral and cognitive symptoms are related to three frontal behavioral categories: apathy, executive dysfunction, and disinhibition. The combination of these symptoms is sometimes referred to as “executive dysfunction syndrome”. All of these symptoms are related to deficits in the cortico-striatal circuits involving the orbitofrontal cortex, dorsolateral prefrontal cortex and anterior cingulate cortex. As discussed above, neuropathological studies have observed a gradual degeneration of the striatum in a dorsal to ventral direction in HD patients. Although the behavioral and cognitive observations partly agree with a progressive impairment of cortico-striatal circuits, the symptomatic findings appear to be more diffuse than expected based on pathological observations. Onset and progression of behavioral and cognitive symptoms in HD is highly heterogeneous, indicating that damage to striatal regions may be more variable and widespread in early stages of HD than previously thought. This view is supported by evidence from several structural imaging studies (Thieben et al., 2002; Rosas et al., 2005; van den Bogaard et al., 2011).

## Risk taking and pathological gambling behavior in HD

### Pathological gambling

While many people are able to gamble recreationally, it may become an overt problem for some, as they develop pathological forms of this behavior. Pathological gambling is characterized by an excessive urge to gamble despite clear negative financial, personal and professional consequences. It has recently been classified as an addiction in DSM-V, as it closely resembles substance abuse disorders in both diagnostic criteria and neuropathology (van Holst et al., 2010; Clark and Goudriaan, 2012). Pathological gambling will be the first and only “behavioral addiction” recognized within the category “*Addiction and Related Disorders”*. Nevertheless, it should be noted that differences exist between addiction to psychoactive substances and addiction to gambling. First, satisfying craving for psychoactive substances lies in consuming the substance of which the effect is known, while satisfying the craving for gambling may have an uncertain outcome as money may be won or not, unless, it is the act of gambling itself, for instance as an exciting activity. Thus, pathological gambling may be more heterogeneous in this respect with also a more uncertain outcome than substance abuse. It should be noted that outcome variability, including both wins and losses, may be crucial to the development of gambling addiction, as it presents a variable intermittent pattern of reinforcement, which is the most powerful form of instrumental/classic conditioning (Sharpe, 2002; Fiorillo et al., 2003). Second, psychoactive substances may more strongly change activity in the brain and peripheral nervous system than gambling, due to their direct pharmacological activity at several neurotransmitter systems, accelerating thereby addictive processes, making substance abuse a more powerful form of addiction.

The underlying neurobiological mechanisms of gambling are complex and involve many different brain regions and neurotransmitter systems (reviewed in Raylu and Oei, 2002; Goudriaan et al., 2004; Potenza, 2013). Predisposition to addiction has been related to a reduced level of dopamine D2 receptors in the striatum, which function in a feedback loop to inhibit further dopamine release. The resulting hyperactivity of dopaminergic pathways increases sensitivity to reward, motivation, and positive reinforcement of the addictive behavior (Volkow et al., 2002; Di Chiara and Bassareo, 2007). Specific motivational changes that occur when pathological gambling develops include increased motivation to gamble (van Holst et al., 2012) and enhanced attention to gambling-related stimuli (Brevers et al., 2011a,b). In addition, pathological gamblers have reduced cognitive control over behavior in general, as exemplified by decreased performance on response inhibition tasks, increased impulsivity, and a preference for immediate over delayed rewards in neurocognitive tasks (Goudriaan et al., 2004; Brevers et al., 2012a; van den Bos et al., 2013a).

Pathological gamblers perform poorly compared to controls on formal reward-related risky decision-making tasks (e.g., Cavedini et al., 2002; Brand et al., 2005; Brevers et al., 2012b; review: Brevers et al., 2013). This poor performance is independent of whether tasks contain explicit and stable rules for wins and losses such as the Game of Dice Task (Brand et al., 2005) or whether subjects have to learn by trial-and-error which choices are advantageous in the long run, such as the IGT (Cavedini et al., 2002; Brevers et al., 2012b; see Section *Risky Decision-Making by HD Patients on Laboratory Tasks* for details of this task). However, gambling severity was rather correlated with performance on decision-making tasks in which probability of outcome is unknown (IGT) than with tasks with explicit rules (Brevers et al., 2012b). This observation is interesting in view of the fact that in normal subjects the second half of the IGT when subjects have learned task contingencies is akin to tasks with explicit rules. Collectively, these data therefore suggest that in pathological gambling impairments in decision-making may result from both decreased executive control, which is related to more explicit rules, and disturbed reward-punishment (emotional) processing, which is more related to trial-and-error learning to assess long-term value of options (van den Bos et al., 2013a, 2014). In addition, it suggests that disturbances in the latter may be a predisposing factor to escalation of gambling behavior.

From these studies it is clear that neurobiological predisposition for developing pathological gambling behavior involves disturbances in both the associative/cognitive control circuit and the limbic circuit (van den Bos et al., 2013a). As a result, pathological gamblers display reduced cognitive control, increased impulsivity, and increased sensitivity to reward, all of which are aspects of behavioral disinhibition (Iacono et al., 2008). The chance that an individual develops an addiction in its life, however, also depends on many other aspects, such as early-life experiences and environmental risks.

### Pathological gambling in HD: epidemiological evidence

With the increasing amount of possibilities offered by the Internet, there has also been a rise in both legal and illegal online gambling opportunities in recent years. These easily accessible and often uncontrolled gambling activities may pose a risk to anyone who has an increased susceptibility to gambling addiction, but may otherwise not become involved in such activities (Griffiths, 2003). HD patients are one of the groups for which Internet gambling may pose such a risk, because behavioral disinhibition—a common feature in the disease—is an important factor in the development of addictions (Iacono et al., 2008). Indeed, as mentioned above, HD patients show several signs of disinhibition, such as irritability, impaired response inhibition, and reduced emotional recognition, at an early stage in the disease. Other symptoms that have been observed in HD, and can influence patients’ ability to make rational decisions, are cognitive inflexibility, perseveration, poor judgment, and reduced self-awareness. Besides these symptomatic similarities between HD patients and pathological gamblers, both groups display structural and functional abnormalities in similar cortico-striatal circuits.

In view of these similarities between pathological gamblers and HD patients, we may expect the incidence of gambling problems to be increased among HD patients compared to the normal population. Nevertheless, only one study so far has reported cases of pathological gambling in an Italian family with HD (De Marchi et al., 1998). In this family, two individuals were diagnosed with pathological gambling around the age of 18, well before the onset of clinical signs of HD. Other epidemiological studies have not reported on this issue, although impaired decision-making, risk taking, and poor judgment have been shown to pose a risk for HD patients handling important life decisions and financial affairs (Klitzman et al., 2007; Shannon, 2011). Similarly, reports on related issues such as substance abuse and addiction to Internet use are missing in the current literature on HD pathology. At this moment, it is unclear whether the absence of reports of gambling problems in the HD literature is caused by a lack of attention for this phenomenon, or whether there really is no increased prevalence of pathological gambling among HD patients. Several reasons may explain why such problems have not been reported more frequently. Firstly, even if the incidence of pathological gambling is increased in HD, this will likely still only affect a small percentage of patients. In combination with the fact that the HD-affected population itself is limited in number, this may cause gambling problems to go unnoticed as a specific issue in this patient group. Secondly, the lack of gambling problems in HD may be related to the inability or unwillingness of patients to leave the house due to motor disorders and frequently observed signs of apathy and depression. Before the advent of Internet gambling, this may have kept HD patients from visiting public gambling places like the casino. Finally, adolescence appears to be a sensitive period for developing gambling problems (van den Bos et al., 2013a), while most HD patients do not start to show disinhibition-related symptoms until later in life. However, with the rise of Internet-related activities of adolescents, they may acquire forms of recreational behavior such as online gambling, which develop into a problem when HD symptoms become manifest later in life. Thus, while the environment in which gambling-susceptible HD patients find themselves may not have promoted such behavior in the past, it is clear that an increased accessibility and availability of gambling opportunities from the home may change the prevalence of related problems in the HD population.

### Risky decision-making by HD patients on laboratory tasks

Laboratory tasks are commonly used to assess cognitive and behavioral abnormalities in neurological disorders. To gain insight into the processes and impairments involved in decision-making and risk-taking behavior, several tasks have been developed, including the IGT (Bechara et al., 1994) and the CGT (Rogers et al., 1999). On the IGT, participants are presented with four decks of cards. They are instructed to choose cards from these decks, with which they can win or lose money; the goal of the task is to win as much money as possible. The decks differ from each other in the frequency and amount of wins and losses. Two of these are “bad” decks, leading to an overall loss in the long run, and two are “good” decks, leading to an overall gain. The participants are not given this information, however, and need to discover which decks are most advantageous during the experiments. Normal, healthy, participants successfully learn the rules of the task after a certain amount of sampling, and eventually start to prefer the two “good” decks. Nevertheless, there are significant individual differences in performance even among healthy participants, including for example clear sex differences (van den Bos et al., 2013b). On the CGT, participants are presented with a row of 10 boxes of two different colors, and need to make a probabilistic decision in which color box a token is hidden. They must then gamble credit points on their confidence in this decision. In this task, all relevant information is presented to the participant during the experiment, and trials are independent, thus minimizing working memory and learning demands. Both gambling tasks are well established, and the IGT is accepted as a valid simulation of real-life decision-making (Buelow and Suhr, 2009), while the CGT is especially useful for studying decision-making outside a learning context.

HD patients have been tested on both the Iowa and Cambridge Gambling Task. In a study with intermediate-stage patients, Stout et al. (2001) found that performance on the IGT was reduced compared to normal subjects. The difference in performance became apparent in the second part of the task; where subjects normally start to show a preference for the good decks, HD patients continued to make frequent selections from the bad decks. This suggests that HD patients either did not learn which decks were advantageous, or continued to choose cards from the bad decks despite this knowledge. The authors noted that several HD participants indicated to know that some decks were disadvantageous, but still continued to select cards from those decks, suggesting that HD patients can learn the rules of the task, but are not able to enforce an advantageous selection pattern and resist responding to individual punishments and rewards. Nevertheless, reduced performance was found to be associated with impaired memory and conceptualization, leading the authors to speculate that HD patients may have trouble learning or remembering the long-term consequences of choosing cards from a particular deck. HD patients also scored higher on disinhibition than healthy controls, but this measure was not correlated with task performance. In a follow-up of the same data Stout and colleagues, compared three cognitive decision models to explain the performance deficit of HD patients, and found that this was best explained by deficits in working memory and by increases in recklessness and impulsivity (Busemeyer and Stout, 2002). Impaired performance of HD patients on the IGT may also be related to a reduced impact of losses on these patients, which was found by measuring skin conductance responses during the IGT (Campbell et al., 2004). This finding is consistent with impaired recognition of negative emotions in HD patients (Johnson et al., 2007; Ille et al., 2011), and suggests that they may be less sensitive to large punishments, and therefore less likely to turn away from the bad card decks. Especially the second part of the IGT requires the ability to suppress disadvantageous courses of action in response to punishments, while reinforcing profitable actions (de Visser et al., 2011; van den Bos et al., 2013b, 2014).

A limited number of other studies have tested risky decision-making in early stages of HD, but did not find performance difficulties in these patients on either the IGT or the CGT (Watkins et al., 2000; Holl et al., 2013). Thus, it appears that impairments in decision-making and risk of gambling problems do not develop until intermediate stages of the disease. However, these studies did find impairments in tasks that required planning and inhibition of pre-potent responses in early HD patients. It thus appears that HD patients first develop subtle problems with inhibition, planning, emotional recognition, and working memory. In some patients this can already lead to problems with judgment and decision-making in early stages of the disease, but most HD patients don’t have problems with risky decision-making tasks until they reach an intermediate stage of the disease.

### Neurobiological mechanisms of decision making in HD

#### Neurobiological pathways underlying normal decision-making processes in the IGT

The neurobiological mechanisms underlying decision-making processes in the IGT have been well studied and described (see e.g., Bechara et al., 2000; Doya, 2008; de Visser et al., 2011; van den Bos et al., 2013b, 2014). Normal execution of this task requires an interaction between the limbic and associative/cognitive control cortico-striatal circuits. Activity in the limbic circuit is thought to be dominant during the first phase of the IGT, during which it is involved in exploratory behavior, responding to rewards and punishments, and learning the affective values of short- and long-term outcomes of decisions in the task (Manes et al., 2002; Clark and Manes, 2004; Fellows and Farah, 2005; Gleichgerrcht et al., 2010; de Visser et al., 2011; van den Bos et al., 2014). The associative/cognitive control circuit, on the other hand, is more important during the second part of the IGT, when it is necessary to suppress impulsive responses to rewards and punishments for long-term benefit, reinforce advantageous behavioral patterns and suppress disadvantageous patterns (Manes et al., 2002; Clark and Manes, 2004; Fellows and Farah, 2005; Gleichgerrcht et al., 2010; de Visser et al., 2011; van den Bos et al., 2014).

#### Neurobiological abnormalities in IGT decision-making processes in HD

Since decision-making processes in the IGT involve an interaction of limbic and associative/cognitive control cortico-striatal circuits, it is not surprising that HD patients are impaired in the performance of this task. One of the observations by Stout and colleagues is that the impact of loss on decision-making is reduced in HD patients (Campbell et al., 2004). This is consistent with findings that these patients are impaired in the recognition of negative emotions, and may be explained by disturbances in the orbitofrontal cortex (Ille et al., 2011). The orbitofrontal cortex is important for emotional processing, and is activated in normal subjects in response to punishments and rewards in a decision-making task (O’Doherty et al., 2001). Another finding by Stout et al. (2001) is that the performance of HD patients on the IGT is correlated with decreased conceptualization and long-term memory measures on the Mattis Dementia Rating Scale. A failure to learn or remember which decks are advantageous on the long-term may be associated with decreased activity of the associative/cognitive control circuit, which is required for long-term planning and impulse control (Manes et al., 2002; Clark and Manes, 2004; Fellows and Farah, 2005; Gleichgerrcht et al., 2010). This is also consistent with specific deficits of the indirect pathway in HD, since a recent study shows that the indirect pathway is important for sensitivity to punishment in a reinforcement-learning task (Kravitz et al., 2012; Paton and Louie, 2012). Insensitivity to the future consequences of a decision may also be caused by ventromedial prefrontal cortex dysfunction, since similar insensitivity is observed in patients with damage to this prefrontal area (Bechara et al., 1994). Thus, decreased performance of HD patients on the IGT may be caused by a combination of dysfunctions in cortico-striatal circuits involving the orbitofrontal cortex, ventromedial prefrontal cortex and dorsolateral prefrontal cortex. This leads to reduced responsiveness to punishment in the first phase of the task, and failure to learn which decks are long-term advantageous, plan accordingly, and suppress impulsive responses in the second phase of the IGT.

## Discussion

### HD and pathological gambling: what are the risks?

The typical array of motor, emotional, and cognitive symptoms of HD is caused by progressive striatal atrophy that affects the different cortico-striatal circuits. Although onset and progression of behavioral and cognitive symptoms appear to be highly heterogeneous, motor and cognitive circuits are typically affected early in the disease, while the limbic circuit is affected at a later stage. Interestingly, neurobiological predisposition to pathological gambling and other addictions involves disturbances in the same cortico-striatal circuits that are affected in HD. Despite these striking similarities, however, in the medical literature HD has not been associated with pathological gambling or other addictive behaviors. Only one study so far has described a family in which gambling problems occurred in several HD-affected family members (De Marchi et al., 1998). We speculate that patients’ motor symptoms, as well as their age and social environment, may thus far have prevented them from developing pathological gambling, despite their increased susceptibility to such problems. On the other hand, the frequently diagnosed depression may be expected to increase impulsivity and the risk of gambling problems, based on correlation studies (Clarke, 2006). Another explanation for the lack of observations of gambling problems in HD may be related to differences in underlying neuropathology. While the cognitive disturbances appear to be highly similar between pathological gamblers and HD patients, the emotional changes are of a different nature. Pathological gamblers mainly show an increased sensitivity to rewards, urging them to start and continue gambling. HD, on the other hand, has been associated with a decreased sensitivity to punishments and negative emotions. This difference may be an important reason why HD patients do not appear to have an increased tendency to start gambling or engage in other rewarding, addictive behaviors.

Nevertheless, disturbances in the limbic cortico-striatal circuit of HD patients may still promote risky decision-making in situations with uncertain outcome, as demonstrated in the IGT (Doya, 2008). Moreover, the combination of decreased sensitivity to punishment, failure to inhibit impulsive responses to immediate rewards, and inability to consider long-term delayed rewards and enforce advantageous behavioral patterns accordingly, makes it likely for HD patients to develop gambling problems, when they encounter a situation that promotes such behavior. Characteristic problems of HD patients with strategy shifting and symptoms of cognitive inflexibility and perseveration may contribute to the progression of pathological behavior in these situations. Thus, we propose that HD patients do not have an increased tendency to start gambling or other addictive behaviors inherent to their neuropathology, but that they do have an increased risk of developing an addiction once they engage in gambling. In accordance with this idea, it has been observed that frontal lesion patients become impulsive and often make poor decisions, but that they do not exhibit increased risk-taking behavior (Miller, 1992; Bechara et al., 2000). This suggests that impaired decision-making and risk-taking or -seeking behavior do not necessarily occur together, and that different combinations of limbic and associative/cognitive control circuit disturbances can have different effects on risky-decision making and gambling behavior. Our hypothesis would also explain why HD patients have not been observed to perform worse on the CGT. Since all information about chances and values of wins and losses is available up front in this task, HD patients may not develop disadvantageous strategies, because they are not actively seeking risks. However, this would need to be tested in more advanced disease patients.

If HD patients indeed have an increased risk of developing pathological gambling behavior when presented with the appropriate situation, the rise of easily accessible Internet gambling opportunities may pose a specific risk for this patient group. Even if they do not actively seek out these situations, HD patients are now much more likely to come across gambling opportunities than they were in the past. This is especially true for patients who spend most of their time at home due to their symptoms, where the Internet may be an important means to occupy them. A higher probability of engaging in gambling behavior may therefore cause a disproportionate increase in related problems in the HD population. We suggest that caretakers should be aware of these possible risks, and preferably try to prevent HD patients from engaging in (online) gambling activities. Moreover, we argue that clinicians should regularly assess the risk and prevalence of gambling-related problems in the HD population, to be able to provide appropriate treatment and guidance to patients and caretakers.

### Future directions

Besides epidemiological studies to assess the prevalence of pathological gambling and other addictions in HD, several lines of research can be suggested to increase our understanding of the issues discussed in this paper. First of all, it would be interesting to link performance deficits on the IGT directly to disturbances in cortico-striatal activity in HD patients. To this end, HD patients’ brain activation patterns can be studied with functional magnetic resonance imaging while performing the IGT, and compared to activity in normal subjects. Activity in the striatum, dorsolateral prefrontal cortex and orbitofrontal cortex is expected to be decreased in HD patients during decision-making on the IGT.

To study the behavioral and neurobiological aspects of gambling-behavior in HD in more detail, currently available rodent disease models can be utilized. On a behavioral level, these animals can be expected to show decreased performance on the IGT, similar to human patients. Rodent versions of the IGT are available (review: de Visser et al., 2011) and the involvement of different neuronal structures in these models is well characterized (de Visser et al., 2011; van den Bos et al., 2013a, 2014). Therefore, such experiments are feasible, and can be combined with in-depth analysis of underlying neuronal changes in rodent models of HD using a variety of techniques. Furthermore, with the advent of more ecological valid research methods and tools to assess the development of pathological behaviors, the risk for developing pathological gambling may be studied under (semi)natural conditions in both humans and animals (van den Bos et al., 2013a). Together, these studies of gambling-related symptoms and underlying neuropathology in both human patients and animal models of HD will provide us with a better understanding of the risks related to gambling—and possibly other addictive behaviors—in HD, and improve our ability to provide appropriate treatment and guidance.

## Conflict of interest statement

The authors declare that the research was conducted in the absence of any commercial or financial relationships that could be construed as a potential conflict of interest.
